# Cases of Variola, Treated with Tincture of Iodine, in the Montreal General Hospital

**Published:** 1853-12

**Authors:** 


*Cases of Variola, treated with Tincture of Iodine, in the Montreal General Hospital.*

TABULATED BY JOHN REDDY, M. D., L. R. C. S. I., HOUSE SURGEON.

| Initials, | Age. | Day Tinct. 1st applied. | Character. | Days in Hospital. | Complication. | Remarks, &c. |
|---|---|---|---|---|---|---|
| E. B. . . | 15 | 2 | C. | 30 | Mild Conjunctivitis. | Very severe; slight superficial pitting nearpoint of nose |
| J. J. . . | 25 | 1 | C. | 57 | Furunculus. | Do. do. do. over forehead and side of nose. |
| J. H. . . | 19 | 2 | Coh. | 20 | Diarrhœa. | Not marked. |
| J. G. . . | 17 | 2 | Coh. | 35 | Bronchitis. | Do. |
| J. M. . . | 20 | 1 | Coh. | 15 | Mild Conjunctivitis. | Do. |
| J. M. . . | 21 | 2 | D. | 25 | Acute Conjunctivitis | Do. |
| W. C. . | 40 | 3 | C. | 46 | Hemorrhoids. | Slightly marked upon nose and centre of each cheek. |
| S. H. B. | 19 | 1 | D. | 21 | None. | Not marked. |
| R. W. . . | 17 | 2 | D. | 17 | Bronchitis. | Do. |
| E. C. . . | 3 | 2 | D. | 35 | Do. | Do. |
| R. L. . . | 17 | 1 | D. | 39 | None. | Very severe; 1 or 2 superficial marks upon the face. |
| J. C. . . | 17 | 2 | D. | 23 | Do. | Not marked. |
| L. C. . . | 19 | 2 | Coh. | 33 | Do. | Severe; slight superficial marking. |
| P. McC. | 20 | 2 | D. | 9 | Do. | Not marked. |
| A. McK. | 4 | 2 | D. | 10 | Do. | Do. |
| H. O. . . | 45 | 1 | D. | 35 | Do. | Do. |
| H. B. . . | 20 | 2 | D. | 12 | Do. | Do. |
| A. T. . . | 12 | 1 | D. | 30 | Do. | Do. |
| C. B. . . | 7 | 1 | D. | 23 | Do. | Do. |
| P. B. . . | 2 | 2 | D. | 26 | Do. | Rubbed the face; superficial marking very slight on forehead. |
| H. O'N. | 5 | 1 | D. | 30 | Do. | Not marked. |
| M. A. B. | 9 | 1 | D. | 30 | Do. | Do. |
| M. F. . . | 24 | 1 | D. | 25 | Do. | Verysuperficial on forehead. |
| H. W. . | 36 | 1 | D. | 25 | Furunculus. | Extremelysevere,no marks. |
| D. C. . . | 25 | 2 | Coh. | 45 | Do. | Half the face painted; no difference in the sides. |
| K. H. . . | 20 | 1 | D. | 25 | None. | Not marked. |
| J. F. . . | 5 mon. | 1 | D. | 25 | Do. | Do. |
| F. R. . . | 18 | 2 | D. | 14 | Do. | Do. |
| C. S. . . | 35 | 1 | D. | 14 | Do. | Do. |
| H. S. . . | 43 | 1 | D. | 12 | Do. | Do. |
| J. L. . . | 35 | 2 | Coh. | 12 | Do. | Do. |
| J. W. . . | 22 | 2 | D. | 13 | Do. | Do. |

N. B.—C., Confluent; Coh., Coherent; D., Discreta.

In addition to the foregoing remarks, I have only to add that I am of opinion that the Tincture of Iodine is a powerful ectrotic, and the best I am acquainted with, and removes the variolous itching which is so distressing to the patient.

J. Reddy.

